# Experimental, quantum chemical and molecular dynamic simulations studies on the corrosion inhibition of mild steel by some carbazole derivatives

**DOI:** 10.1038/s41598-017-02446-0

**Published:** 2017-05-26

**Authors:** Henry U. Nwankwo, Lukman O. Olasunkanmi, Eno E. Ebenso

**Affiliations:** 1 Department of Chemistry, School of Mathematical & Physical Sciences, Faculty of Agriculture, Science and Technology, North-West University (Mafikeng Campus), Private Bag X2046, Mmabatho, 2735 South Africa; 2 Material Science Innovation & Modelling (MaSIM) Research Focus Area, Faculty of Agriculture, Science and Technology, North-West University (Mafikeng Campus), Private Bag X2046, Mmabatho, 2735 South Africa; 3 Department of Chemistry, Faculty of Science, Obafemi Awolowo University, Ile-Ife, 220005 Nigeria

## Abstract

Five selected carbazole derivatives, namely carbazole, 3,6-dibromocarbazole, 2-hydroxycarbazole, 1,2,3,4-tetrahydrocarbazole and 9-(2-ethylhexyl)carbazole-3,6-dicarboxaldehyde were investigated for their inhibitive effects on *Desulfovibrio vulgaris* (*D*. *vulgaris*) induced corrosion of mild steel and in 1 M HCl medium using weight loss, potentiodynamic polarization and electrochemical impedance spectroscopy (EIS) techniques. The carbazole derivatives were found to be mixed type inhibitors with predominantly cathodic inhibitive effects for mild steel in 1 M HCl. Surface morphology results showed the compounds formed adsorbed film on mild steel surface in both aqueous acid and sulphate reducing bacteria (SRB) media. Quantum chemical calculations were used to provide molecular based explanations for the inhibitive effects of the compounds. The interactions of the molecules with mild steel surface was simulated based on molecular dynamic simulations approach using Fe(110) crystal surface as representative metallic surface.

## Introduction

Mild steel is a widely used alloy of iron with various industrial applications. The choice of mild steel as a preferred material for construction and other usages is attributed to its relatively low cost and high mechanical strength^1^. However, mild steel readily undergoes corrosion in common environments of usage. Acid solutions especially hydrochloric acid used in many industrial practices such as acid cleaning, oil-well acidizing, acid descaling etc. are typical highly aggressive media for mild steel corrosion^1^. Corrosion can also be induced by microorganisms. Such corrosion is called microbial influenced corrosion (MIC). It has been suggested that biocorrosion (MIC) follows the same mechanism as electrochemical corrosion in aqueous media^2, 3^.

Though both corrosion in aqueous environment and the MIC are destructive, the former is by far more investigated than the latter. Research in corrosion especially with respect to the use of corrosion inhibitors is often conducted in aqueous environment. A large number of these studies have been carried out on mild steel corrosion in aqueous acid solutions^4–7^. Meanwhile, a study conducted by Rajasekar *et al*.^8^ revealed that about 20% of corrosion damages are due to MIC. Miller *et al*.^9^ also reported that the cost of damages due to MIC stood at nearly 50% of all corrosion cost, which amounted to 140 billion USD in the US alone. In this regard, more studies that focus on MIC or more inclusive studies that encompass both acid corrosion and MIC should be promoted. Formation of biofilms, their characteristics, and influence on bacteria populations have been extensively discussed^10–13^. The effects of anaerobic bacteria on MIC and the contributions of sulphate reducing bacteria (SRB) to MIC can be found in literature^14^.

Various techniques used in the study of corrosion and corrosion inhibition in acid solution include electrochemical^4, 15–18^, quantum chemical^19–22^ and surface morphology^4, 21, 22^. On the other hand, weight loss technique^23, 24^, electrochemical methods^24–27^ and surface analyses techniques^26, 28^ have recently been applied in MIC studies. Various organic compounds, especially those that contain N, O, S, and P heteroatoms as well as π-electron systems have been used as corrosion inhibitors for metals in aqueous solutions^29^. However, only few studies have been reported on the potentials of carbazoles (CZs) as corrosion inhibitors in (aqueous) chemical and/or biological environments. Among the few studies that have expressed the potentials of carbazoles as corrosion inhibitors is the work of Wang *et al*.^30^, in which the corrosion inhibition properties of carbazole and *N*-vinylcarbazole against copper in 0.5 M NaCl solution was investigated using electrochemical techniques. Gopi^31^ reported newly synthesized poly(*N*-vinylcarbazole-co-glycidyl methacrylate) as coating material on low nickel stainless steel, while Abdallah *et al*.^32^ studied the synergistic effects of some halide ions on the inhibition of zinc corrosion in hydrochloric acid by tetrahydrocarbazole derivatives. Despite the huge number of carbazole derivatives, only a few of them have been tested as corrosion inhibitors, while studies on their metal protection potentials against MIC are very scanty.

The present study therefore investigates the adsorption of some carbazole derivatives on mild steel in chemical (aqueous acid) and biological environments and inhibition of steel corrosion in those environments. Five carbazole derivatives were investigated and their corrosion inhibition potentials for mild steel corrosion in microbial environment and 1 M HCl solution were evaluated. The five carbazoles considered in this study include unsubstituted (parent) carbazole and additional four substituted derivatives. The motive was to compare the corrosion inhibition potentials of the derivatives with the parent compound (unsubstituted carbazole), in order to observe the effects of the substituent groups on corrosion inhibition property of carbazole. The inhibitive potentials of the CZs on mild steel corrosion in 1 M HCl were determined using electrochemical techniques. Weight loss method was used to quantify their protection efficiencies for mild steel in *D*. *vulgaris* influenced bio-corrosion. Surface morphology studies using the scanning electron microscope (SEM) and energy dispersive X-ray (EDX) equipment were conducted to ascertain that the studied CZs protect the steel surface against (bio)corrosion in the studied media. Quantum chemical calculations and molecular dynamic simulations were carried out to provide theoretical based explanations for the inhibitive behaviour of the studied carbazoles. The carbazole derivatives investigated in this study have not been tested as corrosion inhibitors for mild steel in MIC as well as 1 M HCl medium in any previous study.

## Experimental Details

### Materials and reagents

The five carbazole derivatives used as corrosion inhibitors in the study were obtained from Sigma-Aldrich and used without further purification. The molecular structures, IUPAC names and adopted abbreviations of the studied CZs are presented in Table 1. The atom numberings in Table 1 are used for discussion of results in the later part of the manuscript. Absolute ethanol 99.8%, Tin(II) chloride dihydrate and glutaraldehyde solution (25%) were supplied by Sigma-Aldrich; hydrochloric acid and acetone (Merck, Modderfontein, South Africa); antimony trioxide from Acros. The chemicals were used without further purification.

**Table 1:** Molecular structures, IUPAC names and abbreviations of the studied CZs.

Structure of inhibitor	IUPAC name	Abbreviation
	Carbazole	CZ
	3,6-dibromocarbazole	DBCZ
	2-hydroxycarbazole	HCZ
	1,2,3,4-tetrahydrocarbazole	THCZ
	9-(2-ethylhexyl)carbazole-3,6-dicarboxaldehyde	EHCZDCA

### Aggressive solutions

The aggressive blank solution of 1 M HCl was prepared by diluting the analytical grade 32% with distilled water. The stock solutions of the inhibitors were prepared using 1:1 mixture of acetone and ethanol as a co-solvent at a quantity of 2.4% (by volume) of the solution. The inhibitor concentrations; 100, 150, 200, 300, 400 and 500 ppm were prepared from the stock solutions.

### SRB culture

The sulphate-reducing bacterium *Desulfobrio vulgaris* (*D.* *vulgaris*, ATCC, No. 7757) was purchased from Leibniz Institute DSMZ-German Collection Microorganisms and Cell Cultures. The composition of the SRB culture is 2.0 g of MgSO_4_·7H_2_O, 5.0 g of sodium citrate, 1.0 g of CaSO_4_·2H_2_O, 1.0 g of NH_4_Cl, 0.5 g of K_2_HPO_4_, 3.5 g of sodium lactate, 1.0 g of yeast extract in 1000 ml of distilled water. The final pH of the SRB culture medium prior to MS incubation was 7.4. The pre-cultures of SRB strains were grown on H_2_ and subsequently flushed with N_2_ for 30 min to prevent contamination by dissolved sulphide and H_2_ into the incubations.

### Electrodes and reagents

Mild steel coupons with percentage weight compositions 0.17% C, 0.46% Si, 0.017% S, 0.019% P, and the remainder iron was used for both aqueous electrochemical corrosion and biocorrosion weight loss tests. Mild steel coupon was cut into 1 cm × 1 cm. The mild steel coupons for electrochemical measurement were embedded in a Teflon holder using epoxy resin, exposing a surface area of 1 cm^2^. Prior to each measurement, mild steel surface was mechanically abraded on Struers MD Piano^TM^ 220 (size: 200 dia) mounted on Struers LaboPol-1 machine to remove traces of epoxy resin from the surface. The surface was then ground using progressively finer SiC papers of grit size 200, 300, 400 and 600 to achieve a mirror-like surface. The coupons were then cleaned in a sonicating acetone bath for 30 mins, followed by sonication in ethanol and finally wiped with clean paper towel and air-dried. The mild steel was used immediately after surface pre-treatment.

### Electrochemical measurements

All electrochemical measurements were carried out using the Autolab PGSTAT 302 N obtained from Metrohm and equipped with a three-electrode glass cell system. Mild steel, platinum rod and Ag/AgCl with 3 M KCl were used as working, counter and reference electrodes respectively. The electrochemical system was maintained in an unperturbed state for a period of 30 minutes in order to allow it to reach the steady open circuit potential (OCP) before each electrochemical measurement.

Potentiodynamic polarization (PDP) measurements were carried out after 30 minutes of mild steel immersion in the aggressive solutions by sweeping the potential between −0.25 to + 0.25 V at the scan rate of 0.5 mVs^−1^. The corrosion current density (*i*_corr_), anodic and cathodic Tafel slopes (b_a_ and b_c_ respectively) were obtained from the polarization curves by extrapolating the linear Tafel segments to the corrosion potential (E_corr_). The inhibition efficiency (%*IE*_PDP_) was calculated from the *i*_*corr*_ using the formula:

1 $$I{E}_{PDP}=100(1-\frac{{i}_{corr}}{{i}_{corr}^{0}})$$

where *i°*_*corr*_ and *i*_*corr*_ are corrosion current densities in the absence and presence of inhibitors respectively.

Electrochemical impedance spectroscopy (EIS) measurements were carried out at the OCP by analysing the frequency response of the electrochemical system in the range of 10 mHz to 100 kHz at 10 mV root-mean-square (rms) amplitude. The impedance spectra were fitted into appropriate equivalent circuit to obtain relevant electrochemical parameters including the charge transfer resistance (R_ct_). The corrosion inhibition efficiency was evaluated from *R*_*ct*_ values using the formula:

2 $$I{E}_{EIS}=100(\frac{{R}_{ct}-{R}_{ct}^{0}}{{R}_{ct}})$$

where *R*_*ct*_ and *R* ^*0*^_*ct*_ are the charge transfer resistances in the presence and absence of inhibitor respectively.

All the electrochemical measurements were carried out under unstirred conditions at 303 K. The measurements were taken in triplicate and the results were adequately reproducible.

### Biocorrosion incubations

The surface pre-treated mild steel coupons were used as the substratum for biofilm growth in 30 ml cylindrical airtight containers. The mild steel was subjected to *D*. *vulgaris* activity in the airtight containers. The steel sample used for surface morphology study was exposed at only one side, while the other sides were coated with Teflon. An anaerobic chamber with a constant nitrogen supply was used to provide an anaerobic environment for inoculation. After distributing 10 ml of the SRB medium into each airtight containers and adding an appropriate amount of biocide, it was inoculated with SRB culture. Bacteria suspensions of 1.5 × 10^8^ CFU/mL were used during the experiments. Prior to inoculation of the samples, the chamber and containers were flushed with steady supply of nitrogen gas to ensure oxygen-free environment. Each container had five metal coupons carefully placed to avoid contact with each other. The containers were sealed, put in an airtight vessel, placed in an incubator maintained at 37 ° C for a period of 9 days.

### Weight loss analysis

Corrosion rates due to the activities of *D*. *vulgaris* were determined by weight loss analysis using ASTM method G01-03^33–35^. Prior to immersion of mild steel, the mirror like coupons was cleaned in a sonicating acetone bath for 30 mins, followed by sonication in ethanol. Mild steel coupons were further sterilized by soaking in ethanol for 48 hrs and later dried in vacuo. The weight of the mild steel was then recorded before exposure to *D*. *vulgaris*. The sterilized mild steel coupons were exposed to *D*. *vulgaris* for a period of 9 days, after which they were retrieved, rinsed in distilled water, wire brushed, and immersed in Clark’s reagent (1000 mL, 12.1 M HCl, 20 g antimony trioxide, and 50 g tin chloride) for 30 seconds to remove surface oxides, biofilm and other corrosion product. Clarke’s solution has been reported to be effective in removing base metal that accumulates on the surface of metallic coupon^28^. The coupons were further rinsed with distilled water, air dried and weighed. Washing (in Clark’s solution and distilled water), drying and weighing were repeated until a stable weight was recorded, indicating complete removal of biofilms and corrosion products.

The weight loss experiment was carried out in triplicates and average weight loss was recorded in each case to ensure reproducibility.

Corrosion rate was calculated using the equation:

3 $$CR=\frac{\Delta W}{AT}$$

where *CR* is the corrosion rate (g.cm^−2^.d^−1^); *ΔW* is the average weight loss (g) of mild steel coupons; *A* is the surface area (cm^2^) and *T* is the duration of immersion in days. From the calculated *CR* values, inhibition efficiency (%*IE*_WL_) was derived using the relationship^36^:

4 $$ \% I{E}_{WL}=\frac{C{R}_{1}-C{R}_{2}}{C{R}_{1}}\times 100$$

where *CR*_1_ and *CR*_2_ are the corrosion rate values in the absence and presence different concentrations of CZs, respectively.

### Surface analysis

For corrosion tests in acidic medium, mild steel specimens with freshly pre-treated surface as described above were immersed in 1 M HCl in the absence and presence of 100 ppm of the studied CZs. Thereafter, the specimens were removed, washed with distilled water, dried and utilized for surface electron microscopy (SEM) and energy dispersive x-ray (EDX) analyses. For biocorrosion study, the steel specimens immersed in the *D*. *vulgaris* incubator for 9 days were used. The retrieved coupon whose surface is covered with biofilm was immersed in 2.5 wt% glutaraldehyde for 8 h and subsequently washed with a graded series (30%, 50%, 70%, 100% v/v) of ethanol for dehydration^34, 37^, and finally stored in a desiccator and later used for SEM and EDX analyses. The SEM images with simultaneous elemental characterization using EDX were obtained using FEI Quanta FEG 250 Environmental Scanning electron microscope (ESEM) under an acceleration voltage of 15 kV in high vacuum.

### Quantum chemical calculations

Quantum chemical calculations were carried out using the density functional theory (DFT) method comprising the Becke three-parameter hybrid functional together with the correlation functional, which includes both local and non-local terms (B3LYP), and the split-valence double-zeta polarized basis set (6–31 G + (d, p))^25^. Full geometry optimizations were carried out on neutral molecules both in vacuo and in water phases. Calculations were not performed for protonated species because it has been reported that protonation of carbazoles in acidic medium might require rigorous conditions, such as highly concentrated acid^38^. Polarizable continuum model was used to investigate the effect of water on the geometry and electronic parameters of the molecules. Quantum chemical parameters computed include the energy of the highest occupied molecular orbital (*E*_*HOMO*_), the energy of the lowest unoccupied molecular orbital (*E*_*LUMO*_), energy band gap (Δ*E* = *E*_*LUMO*_ − *E*_*HOMO*_), global electronic chemical potential (*µ*), chemical softness (*σ*), chemical hardness (*η*) and electrophilicity index (*ω*). The quantum chemical reactivity indices were derived from the frontier molecular orbital energies (*E*_*HOMO*_ *and E*_*LUMO*_) using appropriate relations (Equations 5–9) as previously reported in literature^39^.

5 $$\Delta E={E}_{LUMO}-{E}_{HOMO}$$

6 $$\eta =-\frac{1}{2}({E}_{HOMO}-{E}_{LUMO})$$

7 $$\sigma =\frac{1}{\eta }=-(\frac{2}{{E}_{HOMO}-{E}_{LUMO}})$$

8 $$\mu =\frac{1}{2}({E}_{HOMO}+{E}_{LUMO})$$

9 $$\omega =\frac{{\mu }^{2}}{2\eta }$$

The quantum chemical studies were carried out with the aid of Gaussian 09 software^40^. In order to identify the prospective local (atomic) sites of the inhibitor molecules that are susceptible to electrophilic attacks by the metallic atoms, the electron density surfaces of the electrophilic Fukui function (*f* ^−^) of the studied CZs were visualized using the Multiwfn software^41, 42^.

### Molecular dynamics simulation

Molecular dynamic simulation was carried out to describe the interaction between the inhibitor molecules and metallic surface. The adsorption locator module implemented in the Materials studio 6.0 software from Accelrys was used for the simulation. The inhibitor molecules were modelled and optimized using the Condensed-phase Optimized Molecular Potentials for Atomistic Simulation Studies (COMPASS) force field. COMPASS is a robust and well-developed force field that was derived based on fitting against a wide range of experimental data for organic and inorganic compounds^43^. This informs its suitability for treating metal and non-metal containing systems. Fe (110) crystal surface was used as the representative metallic surface. The Fe (110) crystal surface was obtained by cleaving the crystal structure of Fe into 110 planes and optimizes the surface to minimum energy using the COMPASS force field. A 10 × 10 supercell of Fe (110) was built from the optimized crystal surface and a vacuum slab of 7 nm was built above the plane.

The optimized inhibitor molecule was used as the adsorbate and made to interact with the surface atoms of the Fe (110) using the adsorption locator module. A maximum distance of 10 Å was maintained between the adsorbate and the selected atoms of Fe(110) surface. Ultra-fine convergence criteria was used for all optimization and simulation jobs, while the temperature was ramped down from 1 × 10^5^ K to 100 K as the simulations went through 10 cycles at 100,000 steps per cycle.

## Results and Discussions

### Electrochemical measurements

#### Potentiodynamic polarization (PDP) studies

**Figure 1:**
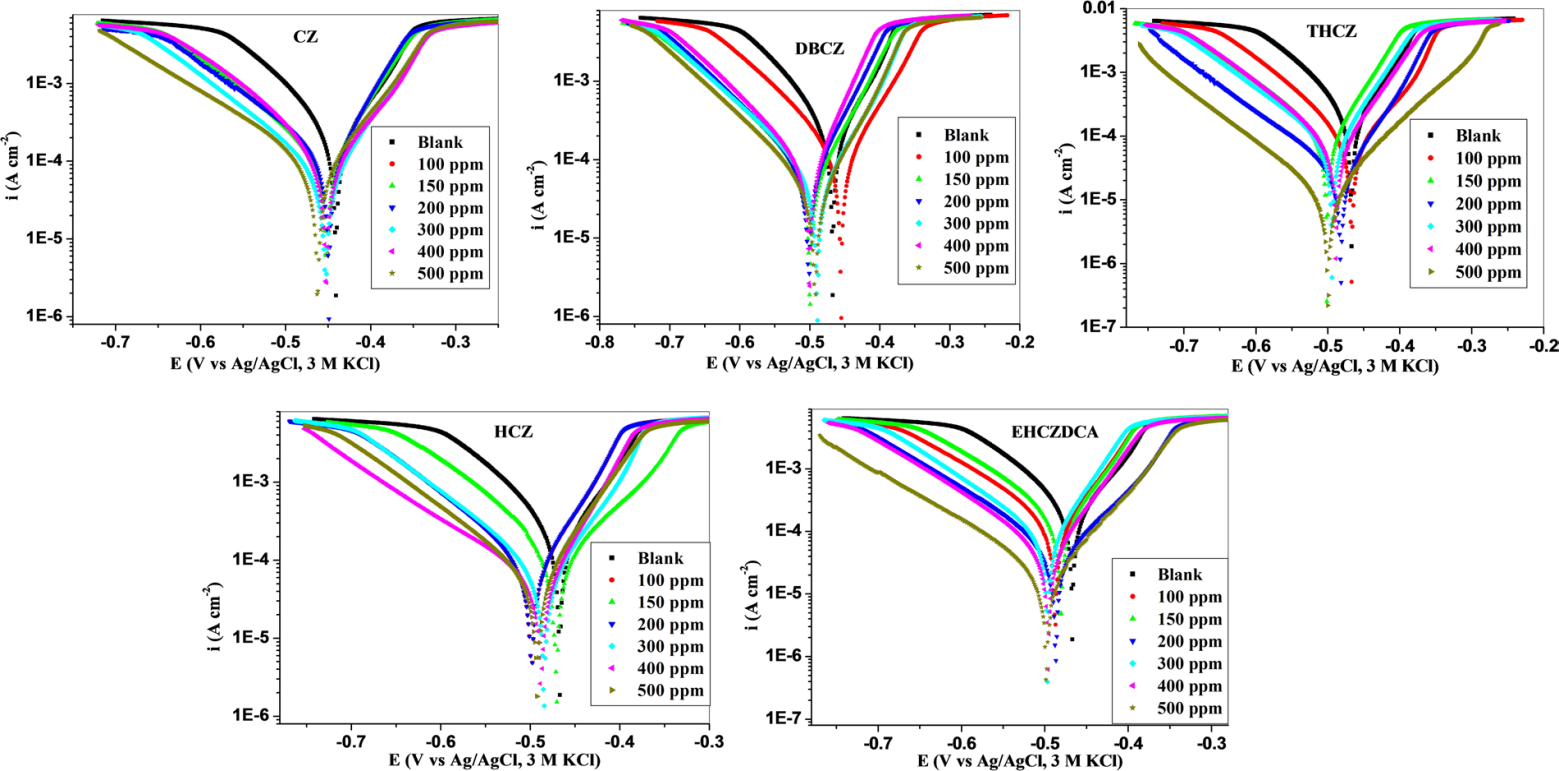
Polarization curves for mild steel in absence and presence of different concentrations of CZs.

Potentiodynamic polarization studies were carried out to gain insights into the mechanism of the mild steel dissolution at the anode and evolution of hydrogen at the cathode in the absence and presence of different concentrations of CZs. The current-potential curves for mild steel corrosion in 1 M HCl without and with different concentrations of the CZs are shown in Fig. 1.

The polarization curves in Fig. 1 clearly reveal that the addition of the inhibitors affects both the anodic and cathodic processes as the curves are shifted towards lower corrosion current density region compared to the blank. The reduction in anodic and cathodic current densities increases as inhibitor concentration increases. This implies that the corrosion rate decreases with increasing concentration of the inhibitors and the inhibitors retard the rate of both anodic and cathodic corrosion reactions^44^. The shifts in corrosion current densities appear to be more apparent for the cathodic curves than the anodic. This suggests that the inhibitors might have more pronounced effects on the cathodic reaction than the anodic one. More so, cathodic Tafel lines exhibit near parallel displacements with increasing concentration of the inhibitors, which suggests that the hydrogen gas evolution is activation-controlled and addition of inhibitors did not affect the mechanism of this process^45, 46^. The similar behaviour of the polarization curves both in the absence and presence of inhibitors hints that addition of the inhibitors did not alter the corrosion mechanism.

Relevant electrochemical parameters such as corrosion current density (*i*_corr_) and anodic and cathodic Tafel slopes (*b*_***a***_ and *b*_*c*_ respectively) were obtained by extrapolating the linear Tafel regions of the polarization curves to the E_corr_. The results of the electrochemical parameters together with the inhibition efficiencies are presented in Table 2. The E_corr_ values for the inhibited systems are more cathodic compared to the blank, except for 100 ppm DBCZ, which is slightly more anodic. This observation further supports the suggestion that the inhibitive effects of the studied compounds are more profound on the cathodic reaction.

**Table 2:** Tafel polarization parameters for MS in 1 M HCl solution in absence and at different concentration of carbazoles.

Inhibitor	Conc (ppm)	*b*_*a*_ (mV/dec)	*b*_*c*_ (mV/dec)	*−Ε*_corr_ (μς)	*i*_corr_ (*μ*A cm^−2^)	*η*%
Blank	—	98.98 ± 1.63	72.03 ± 0.82	452.91 ± 2.45	236.07 ± 2.04	—
CZ	100	123.75 ± 1.02	69.18 ± 1.58	497.80 ± 1.22	133.63 ± 1.45	43.39 ± 0.60
	150	130.55 ± 1.10	63.02 ± 0.14	500.26 ± 0.83	129.70 ± 0.93	45.06 ± 0.51
	200	137.05 ± 0.71	80.27 ± 0.56	505.95 ± 1.72	114.25 ± 1.02	51.60 ± 0.64
	300	107.39 ± 1.27	87.41 ± 0.91	433.62 ± 0.60	111.17 ± 0.97	52.91 ± 0.65
	400	144.94 ± 0.87	77.47 ± 1.21	478.54 ± 1.27	84.46 ± 0.78	64.22 ± 0.82
	500	56.65 ± 1.14	96.62 ± 1.24	493.23 ± 1.02	60.42 ± 1.20	74.41 ± 1.61
DBCZ	100	124.18 ± 0.99	79.70 ± 1.40	440.76 ± 1.56	141.88 ± 1.94	39.90 ± 0.65
	150	125.43 ± 1.22	80.36 ± 1.46	482.89 ± 0.74	103.72 ± 1.75	56.06 ± 1.06
	200	124.25 ± 1.10	67.24 ± 1.41	490.84 ± 2.12	88.37 ± 2.02	62.57 ± 1.53
	300	134.98 ± 0.97	78.26 ± 0.95	486.05 ± 0.96	86.04 ± 0.91	63.55 ± 0.87
	400	54.66 ± 1.37	110.41 ± 2.06	498.77 ± 2.18	78.45 ± 1.80	66.77 ± 1.63
	500	124.80 ± 1.53	68.77 ± 2.06	482.55 ± 1.27	51.18 ± 1.06	78.32 ± 1.76
HCZ	100	118.57 ± 1.33	64.35 ± 0.44	481.27 ± 0.96	102.65 ± 0.45	56.52 ± 0.55
	150	94.46 ± 1.05	83.61 ± 1.13	449.77 ± 1.81	85.24 ± 0.92	63.89 ± 0.89
	200	65.93 ± 1.44	121.99 ± 1.18	480.15 ± 1.09	82.04 ± 0.77	65.25 ± 0.83
	300	144.64 ± 1.27	56.71 ± 1.22	489.62 ± 1.33	57.74 ± 1.58	75.54 ± 2.17
	400	133.06 ± 0.94	64.09 ± 0.76	491.49 ± 1.83	53.51 ± 1.32	77.33 ± 2.02
	500	60.00 ± 1.46	99.82 ± 2.23	492.14 ± 1.85	46.17 ± 1.99	80.44 ± 3.54
THCZ	100	123.25 ± 1.03	87.50 ± 1.48	484.01 ± 0.83	140.20 ± 2.41	40.61 ± 0.78
	150	55.66 ± 2.12	111.29 ± 2.16	495.39 ± 1.24	82.23 ± 2.32	65.17 ± 1.92
	200	159.60 ± 1.32	75.19 ± 0.93	481.00 ± 1.45	68.17 ± 1.58	71.12 ± 1.76
	300	58.50 ± 1.71	121.49 ± 1.16	488.13 ± 1.91	68.40 ± 3.27	71.03 ± 3.46
	400	59.53 ± 2.01	85.81 ± 1.38	487.35 ± 4.21	46.74 ± 1.68	80.20 ± 2.97
	500	119.46 ± 2.23	92.87 ± 2.11	505.37 ± 2.19	12.77 ± 1.84	94.59 ± 13.69
EHCZDCA	100	116.50 ± 1.54	64.23 ± 1.00	472.42 ± 1.89	129.45 ± 2.76	45.16 ± 1.04
	150	85.54 ± 0.32	58.28 ± 0.27	476.72 ± 0.48	114.62 ± 0.53	51.45 ± 0.50
	200	11.46 ± 1.71	56.71 ± 2.07	492.41 ± 1.31	83.67 ± 1.59	64.56 ± 1.35
	300	129.79 ± 2.07	85.22 ± 1.17	482.57 ± 1.53	70.99 ± 2.30	69.93 ± 2.35
	400	54.08 ± 0.30	107.58 ± 1.18	491.63 ± 1.98	41.59 ± 1.19	82.38 ± 2.47
	500	125.62 ± 1.58	70.18 ± 1.31	490.80 ± 2.54	21.51 ± 1.13	90.89 ± 4.86

The maximum shift in *E*_corr_ with respect to the E_corr_ of the blank is 53 mV (observed for CZ at 200 ppm and THCZ at 500 ppm). A shift in E_corr_ less than 85 mV is generally attributed to mixed-type inhibitive effect of a corrosion inhibitor^36^. Therefore, it can be inferred that the studied CZs are mixed-type inhibitors with predominant effects on cathodic reaction. That is, the inhibitors retard both the anodic mild steel dissolution and the cathodic hydrogen evolution reactions with the latter being more affected. The change in cathodic Tafel slopes, b_c_ with change in inhibitors concentration is an indication of the effects of the inhibitors on the kinetics of the cathodic process. The change in anodic Tafel slopes, b_a_ on the other hand is attributable to the adsorption of species such as chloride ion or inhibitor molecules or metal-inhibitor complexes emerging from redox processes, on the active sites on the metal^45, 47^. The inhibition efficiency was found to increase with concentration for the five CZs studied, with the highest %*IE* recorded at 500 ppm for each compound.

#### Electrochemical impedance spectroscopy (EIS) measurements

**Figure 2:**
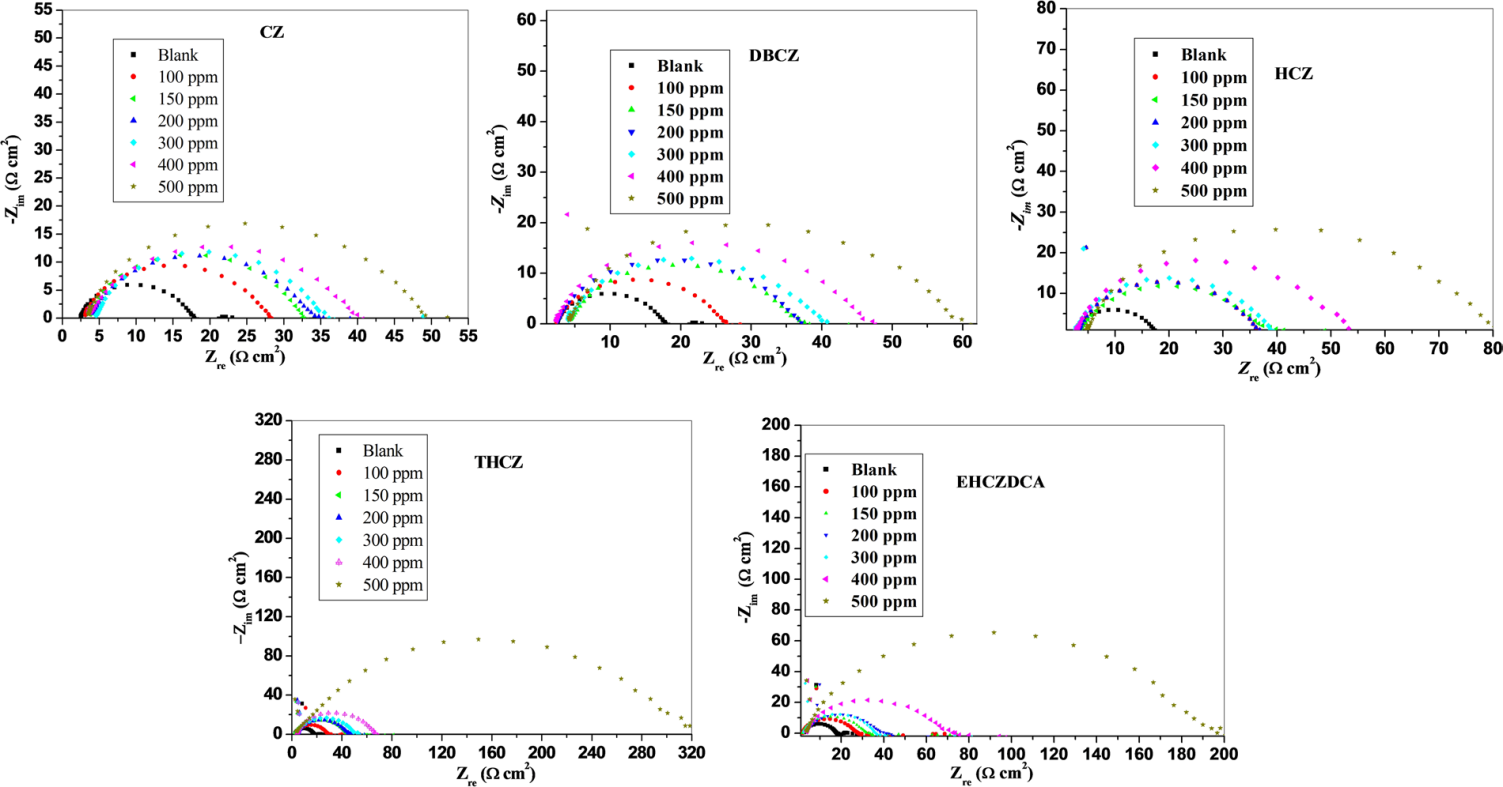
Nyquist plot for mild steel in 1 M HCl in the absence and presence of different concentrations of CZs.

**Figure 3:**
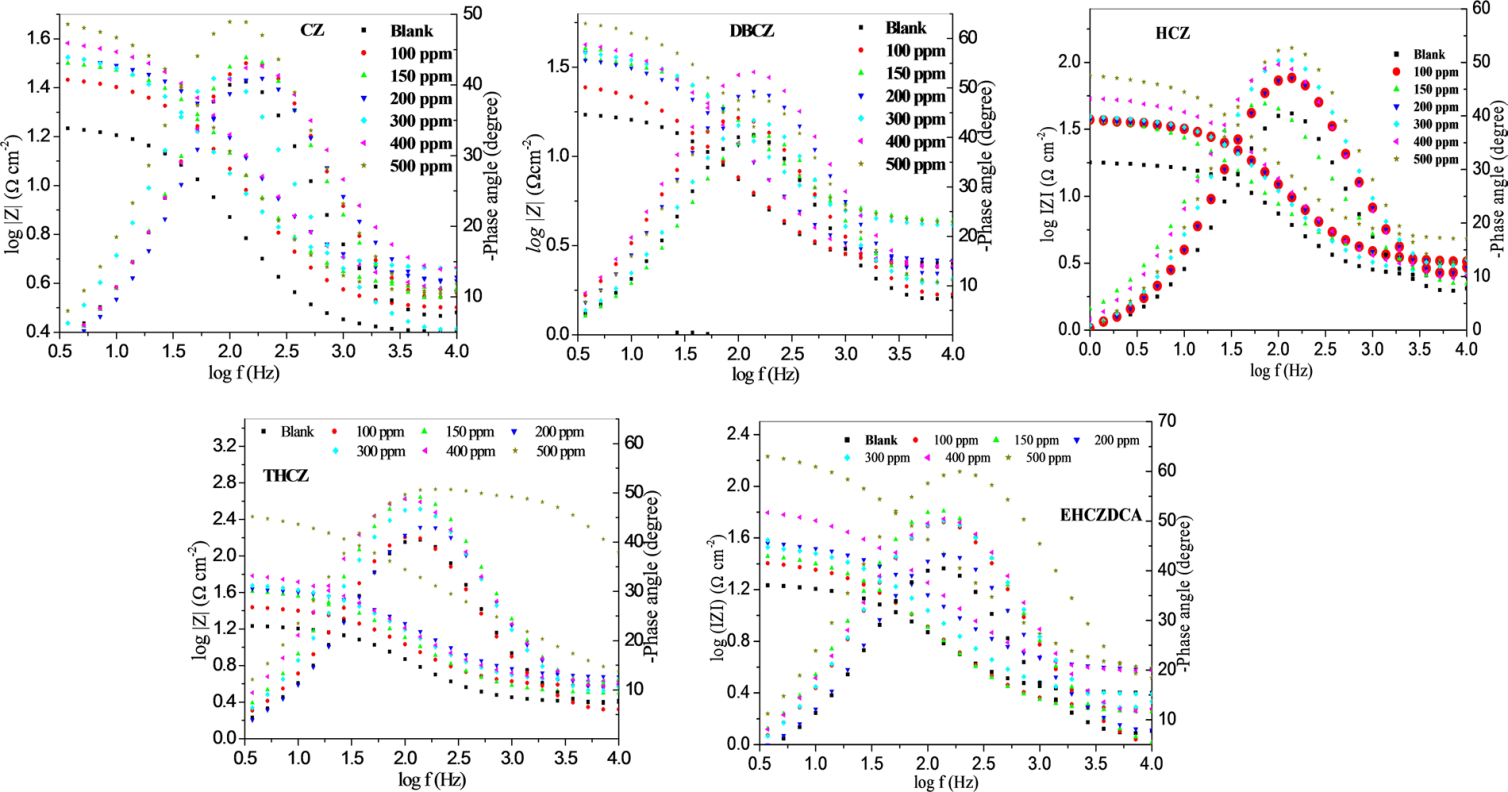
Bode impedance modulus and phase angle plots for mild steel in 1 M HCl in absence and presence of different concentration of CZs.

Electrochemical impedance spectroscopy (EIS) was employed to further elucidate the adsorption of the studied inhibitor molecules on mild steel surface during the electrochemical corrosion reactions occurring at the metal/electrolyte interface^21^. The impedance spectra of mild steel in 1 M HCl in the absence and presence of different concentrations of CZs are presented in Figs 2 and 3 for the Nyquist and Bode plots respectively.

The Nyquist plots show single depressed semicircles with large capacitive loop at low frequency, while the Bode plots show one time constant. This implies that the corrosion of mild steel in the studied media is controlled by single charge transfer process. The depression in Nyquist semicircles is a common feature of solid electrodes which is often referred to as frequency dispersion as a result of the inhomogeneity or roughness of the electrode surface^4, 21^.

Addition of the inhibitors increases the diameter of the capacitive loop of the Nyquist plots without affecting their characteristic features. This means that the inhibitive action of the studied CZs is due to the adsorption of their molecules on the steel surface without affecting the corrosion mechanism^48^. The gradual broadening of phase angle maxima at intermediate frequency in the presence of the studied CZs also suggests the formation of a protective film of the inhibitor molecules on the steel surface^15^.

**Figure 4:**
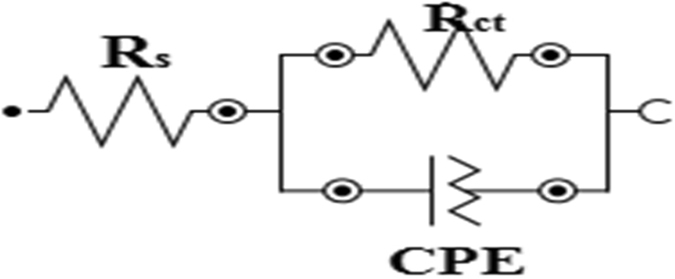
Equivalent circuit employed for the fitting of impedance spectra.

Figure 4 represents the equivalent circuit employed for the fitting of impedance spectra. The constant phase element (CPE), is introduced in the circuit in order to ensure a more accurate description of the process occurring at the electrode/electrolyte interface^48^. The impedance of the CPE (Z_CPE_) is expressed as:

10 $${Z}_{CPE}=(\frac{1}{{Y}_{0}}){[{(j\omega )}^{n}]}^{-1}$$

where, *Y*_*0*_ is the CPE constant; *ω* is the angular frequency; *j* is the imaginary number and *n* is the phase shift (exponent). Electrochemical parameters obtained from the fitting of the impedance spectra are listed in Table 3. The electrochemical behaviour of the CPE is related to the value of *n*, which in turn is related to the surface inhomogeneity^36^. R_ct_ represents the charge-transfer resistance whose value is a measure of electron transfer across electrode/electrolyte interface and it is inversely proportional to corrosion rate^48^. The R_ct_ values in Table 3 increase with increase in concentration of the inhibitors. This implies that the impedance of the mild steel electrode surface to flow of charges across metal-electrolyte interface increases with increasing concentration of the inhibitors. The increased R_ct_ with increase in inhibitor concentration may be as a result of increase in the number of inhibitor molecules that adsorb on mild steel surface, and consequently decrease in the exposed surface area of the mild steel to the aggressive solution. The increased R_ct_ with increase in inhibitor concentration also translates to increase in inhibition efficiency (*IE*%).

**Table 3:** Electrochemical impedance parameters obtained for MS in 1 M HCl in absence and presence of different concentration of carbazoles.

Inhibitor	Conc	*R*_s_	*R*_ct_	Y_0_	*n*	*η*%
	(ppm)	(Ω cm^**−**2^)	(Ω cm^**−**2^)	(*μ*Mh_0_ cm^**−**2^)		
Blank	—	2.50 ± 0.31	16.60 ± 0.73	677.01 ± 4.08	0.84 ± 0.02	—
CZ	100	3.00 ± 0.24	27.90 ± 1.57	487.23 ± 5.00	0.81 ± 0.03	40.50 ± 2.88
	150	3.61 ± 0.34	32.10 ± 1.25	382.45 ± 2.45	0.82 ± 0.03	48.29 ± 2.83
	200	3.70 ± 0.46	34.90 ± 1.98	513.61 ± 3.27	0.75 ± 0.05	52.44 ± 3.76
	300	4.57 ± 0.29	35.10 ± 1.10	553.76 ± 4.08	0.82 ± 0.05	52.71 ± 2.84
	400	3.35 ± 0.10	42.00 ± 2.80	525.09 ± 2.37	0.74 ± 0.04	60.48 ± 4.82
	500	3.37 ± 0.17	51.71 ± 2.12	428.11 ± 1.96	0.81 ± 0.03	67.90 ± 4.07
DBCZ	100	2.38 ± 0.11	25.90 ± 2.04	817.13 ± 4.08	0.80 ± 0.07	35.91 ± 3.24
	150	4.82 ± 0.69	37.02 ± 2.31	454.32 ± 1.81	0.78 ± 0.09	55.16 ± 4.21
	200	2.43 ± 0.23	38.51 ± 1.25	492.21 ± 2.82	0.80 ± 0.10	56.89 ± 3.10
	300	3.78 ± 0.25	43.60 ± 2.12	550.01 ± 4.98	0.75 ± 0.06	61.93 ± 4.05
	400	2.22 ± 0.16	50.51 ± 2.31	485.73 ± 2.62	0.80 ± 0.05	67.14 ± 4.25
	500	4.01 ± 0.11	62.63 ± 1.80	335.30 ± 3.29	0.80 ± 0.09	73.50 ± 3.85
HCZ	100	3.06 ± 0.14	38.42 ± 1.29	472.27 ± 2.47	0.81 ± 0.02	56.79 ± 3.13
	150	3.04 ± 0.23	41.53 ± 2.39	491.33 ± 1.87	0.72 ± 0.06	60.03 ± 4.34
	200	2.50 ± 0.12	42.33 ± 1.22	507.19 ± 1.83	0.81 ± 0.06	60.78 ± 3.18
	300	2.65 ± 0.29	57.71 ± 1.98	404.06 ± 1.31	0.81 ± 0.02	71.24 ± 3.97
	400	4.28 ± 0.34	59.50 ± 2.21	307.20 ± 1.74	0.81 ± 0.03	72.10 ± 4.14
	500	4.52 ± 0.25	87.70 ± 2.87	379.90 ± 2.04	0.79 ± 0.06	81.07 ± 4.43
THCZ	100	3.73 ± 0.28	27.72 ± 2.06	536.17 ± 1.88	0.82 ± 0.02	40.12 ± 3.46
	150	2.97 ± 0.35	46.11 ± 2.64	448.55 ± 4.16	0.81 ± 0.03	64.00 ± 4.61
	200	4.26 ± 0.20	52.91 ± 2.07	550.29 ± 3.53	0.73 ± 0.02	68.63 ± 4.03
	300	3.97 ± 0.38	54.03 ± 1.02	402.14 ± 2.80	0.80 ± 0.04	69.28 ± 3.30
	400	3.99 ± 0.38	71.82 ± 3.75	416.20 ± 1.22	0.78 ± 0.06	76.89 ± 5.24
	500	2.31 ± 0.07	374.01 ± 1.78	186.67 ± 2.96	0.65 ± 0.04	95.56 ± 4.21
EHCZDCA	100	1.87 ± 0.25	28.80 ± 1.68	628.02 ± 2.30	0.83 ± 0.03	42.36 ± 3.09
	150	1.78 ± 0.20	33.73 ± 1.98	624.36 ± 1.98	0.83 ± 0.04	50.79 ± 3.72
	200	2.29 ± 0.23	37.81 ± 1.63	515.09 ± 2.80	0.81 ± 0.04	56.10 ± 3.45
	300	3.64 ± 0.28	42.22 ± 0.75	563.63 ± 4.82	0.76 ± 0.03	60.68 ± 2.87
	400	3.37 ± 0.15	79.41 ± 2.57	416.09 ± 2.46	0.77 ± 0.02	79.10 ± 4.31
	500	3.43 ± 0.13	206.08 ± 3.53	134.11 ± 2.10	0.81 ± 0.06	91.94 ± 4.32

The variations of impedance modulus and phase angles with frequency are visualized in Fig. 3. The impedance at high frequency limit corresponds to the ohmic resistances of the protective film and the solution entrapped between the working electrode and the reference electrode. At high frequencies, log|Z| tends to approach zero and the phase angle (*α*) falls rapidly to 0°^15^. This response is attributed to resistive behaviour of the electrode/electrolyte interface and the impedance magnitude corresponds to the solution resistance. A linear plot of log|Z| vs log *f* with a slope (*S*) of −1 and a phase angle (α) of −90° at intermediate frequencies is characteristic of pure capacitive behaviour of electrode/electrolyte interface^15, 36^. The values of *S* and α as listed in Table 4 shows that the electrochemical systems of mild steel in 1 M HCl in the absence and presence of CZs exhibit pseudo-capacitive behaviour. The values of *S* and α for the inhibitor-containing systems are generally closer to the threshold values for a pure capacitor. This suggests the inhibitor molecules form (pseudo-capacitive) adsorbed film on mild steel surface and thereby protect the steel surface from direct attack by aggressive acidic ions in solution.

**Table 4:** The slopes (*S*) of the Bode impedance modulus plots at intermediate frequencies and the maximum phase angles (*α*) for mild steel in 1 M HCl solution in the absence and presence of 500 ppm inhibitors.

Inhibitor	*−S*	*−α*
Blank	0.30 ± 0.02	40.23 ± 1.28
CZ	0.38 ± 0.02	48.00 ± 1.51
DBCZ	0.52 ± 0.05	48.41 ± 1.32
HCZ	0.42 ± 0.03	49.62 ± 1.92
THCZ	0.39 ± 0.04	50.27 ± 1.07
EHCZDCA	0.54 ± 0.03	58.57 ± 1.30

### Adsorption isotherm

**Figure 5:**
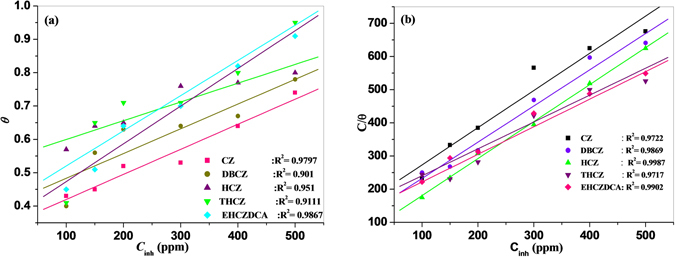
Adsorption isotherm plots of (**a**) Freundlich, (**b**) Langmuir for mild steel in the presence of carbazoles.

Understanding the nature of interactions between the corroding surface of the metal and inhibitor molecules during corrosion inhibition can be expanded in terms of the adsorption characteristics of the inhibitor molecule^49^. In the present study, the results obtained for the degree of surface coverage at 303 K (using the data obtained from polarization studies and listed in Table 2) were fitted into various adsorption isotherm models but only Langmuir and Freundlich adsorption isotherms gave satisfactory fittings with near unity values of the correlation coefficients (R^2^). The general equations for the adsorption models are:

11 $${\rm{Freundlich}}\,\text{isotherm}:\theta ={K}_{ads}{C}_{inh}$$

12 $${\rm{Langmuir}}\,\text{isotherm}:\frac{{C}_{inh}}{\theta }=\frac{1}{{K}_{ads}}+{C}_{inh}$$

where, *θ* is the degree of surface coverage; *K*_*ads*_ is the equilibrium constant of the adsorption/desorption process and *C*_*inh*_ is the concentration of the inhibitor. The value of *K*_*ads*_ is an indicator of the degree of adsorption, i.e., the higher the value of *K*_*ads*_ the stronger the adsorption of the inhibitor molecules on the metal surface^4, 49^. Both the Freundlich and Langmuir adsorption isotherm plots of the forms in Equations 11 and 12 respectively are shown in Fig. 5 for all the studied compounds. The plots exhibit essential linearity with *R* ^2^ values ranging from 0.9717 to 0.9987 for Langmuir, and 0.901 to 0.9867 for Freundlich adsorption isotherms. This suggests a satisfactory level of fitness of the adsorption data to both model. However, the Freundlich adsorption model in Equation 11 expects intercept at θ = 0, while the slope of the Langmuir adsorption isotherm in Equation 12 should be unity. The values of slopes and intercepts listed in Table 5 do not conform to these conditions for the two models. The deviation of the slope of a Langmuir adsorption plot from unity has been attributed to possible interactions between the adsorbed inhibitor molecules^49^, which the actual Langmuir adsorption model does not take into consideration.

**Table 5:** Langmuir and Freundlich parameters for the adsorption of CZs on mild steel surface obtained from Tafel parameters at 303 K.

Isotherm	Inhibitor	Slope	Intercept	K_ads_	−ΔG°_ads_ (kJ/mol)
Langmuir	CZ	1.121	161.435	1035.710	27.610
	DBCZ	1.091	123.656	2628.260	29.960
	HCZ	1.115	69.390	2640.310	29.970
	THCZ	0.831	138.990	1232.040	28.050
	EHCZDCA	0.810	159.779	2099.400	29.390
Freundlich	CZ	7.516 × 10^**−**4^	0.345	—	—
	DBCZ	7.410 × 10^**−**4^	0.410	—	—
	HCZ	5.621 × 10^**−**4^	0.544	—	—
	THCZ	11.000 × 10^**−**4^	0.415	—	—
	EHCZDCA	11.000 × 10^**−**4^	0.361	—	—

A modified Langmuir adsorption isotherm of the form, C_inh_/θ = 1/K_ads_ + mC_inh_ has been used in some recent studies. In this modified model, m is the slope of the plot and can be assumed to an inherent factor in the surface coverage values. Therefore, the intercept of the plots can be equated to (1 /m)K_ads_, from which K_ads_ can be calculated. This approach was adopted to calculate the values of K_ads_ listed in Table 5. Hence, the values of K_ads_ were calculated using the Langmuir adsorption isotherm parameters because there is no similar simple modification for the Freundlich adsorption isotherm.

The change in Gibb’s free energy of adsorption (*ΔG*_*ads*_) was calculated by using the equation:

13 $$\Delta {G}_{ads}=-\,RT\,\mathrm{ln}(55.5{K}_{ads})$$

where *ΔG*_*ads*_ is the standard free energy of adsorption; *R* is the gas constant and *T* is the absolute temperature. The value of 55.5 is the concentration of water in solution in molL^−1^.

The calculated values of *K*_*ads*_ and *ΔG*_*ads*_ are listed in Table 5. Inspection of the results in Table 5 reveals the *ΔG*_*ads*_ values are negative as expected for an adsorption process, which implies spontaneity of the adsorption process and stability of the adsorbed film on the mild steel surface^4, 21^. The *ΔG*_*ads*_ values obtained in this work are more negative than −20 kJmol^−1^ (for physisorption) and less negative than −40 kJmol^−1^ (for chemisorption). This suggests that the studied CZ molecules adsorb on mild steel surface in 1 M HCl via competitive physical and chemical adsorption mechanisms^21^.

### Biocorrosion study (weight-loss measurements)

**Figure 6:**
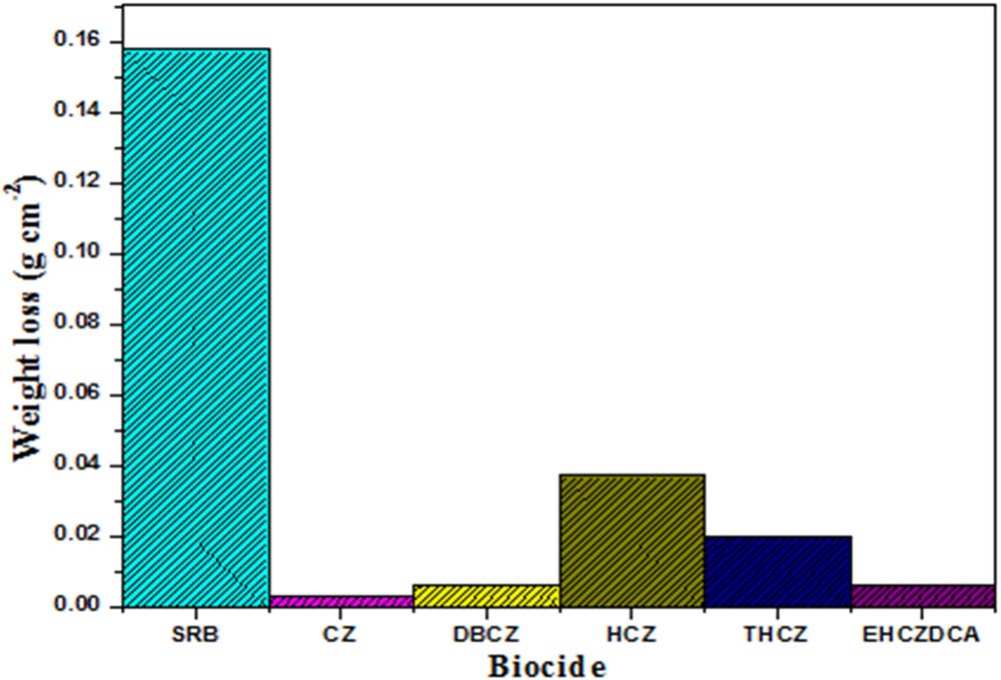
Weight loss measurements for mild steel exposed to SRB cultures after 9 days incubation with or without 100 ppm carbazoles at 310 K.

SRB induced anaerobic corrosion of mild steel was quantified by weight-loss measurements and the degree of weight loss in the absence and presence of the studied inhibitors (CZs) are presented as column plots in Fig. 6. The trend of weight-loss (WL) values for MS exposed to pure and mixed SRB culture is such that SRB > HCZ > THCZ > DBCZ ≈EHCZDCA > CZ. The significant WL observed in pure SRB strain is consistent with the pit image caused by the uninhibited corrosive environment^25^. Corrosion rate (*CR*) due to MIC was also calculated using Equation 3 whilst inhibition efficiency from weight loss analysis (%*IE*_WL_) was calculated using Equation 4 and the results are listed in Table 6. All the studied compounds showed excellent corrosion mitigation efficiencies for mild steel in the SRB culture. The corrosion inhibitive effect of the studied CZs in the SRB media may be as a result of biocidal effect of CZs on the bacteria that are responsible for corrosion in the SRB media. This however does not completely preclude the protection of the steel surface by adsorbed molecules of the tested CZs.

**Table 6:** The weight loss parameters obtained for mild steel exposed to SRB cultures after 9 days incubation with or without 100 ppm CZs.

Biocide	*CR* (g.cm^−2^.d^−1^)	IE%_WL_
SRB	1.97 × 10^**−**2^ ± 2.36 × 10^**−**3^	—
CZ	3.09 × 10^**−**4^ ± 1.69 × 10^**−**5^	98.43 ± 12.96
THCZ	2.17 × 10^**−**3^ ± 1.16 × 10^**−**4^	88.97 ± 11.67
EHCZDCA	9.17 × 10^**−**4^ ± 2.05 × 10^**−**5^	95.34 ± 11.62
HCZ	4.16 × 10^**−**3^ ± 2.76 × 10^**−**4^	78.86 ± 10.81
DBCZ	9.23 × 10^**−**4^ ± 2.70 × 10^**−**5^	95.31 ± 11.76

### Surface morphology studies

#### Scanning electron microscopy (SEM) study

**Figure 7:**
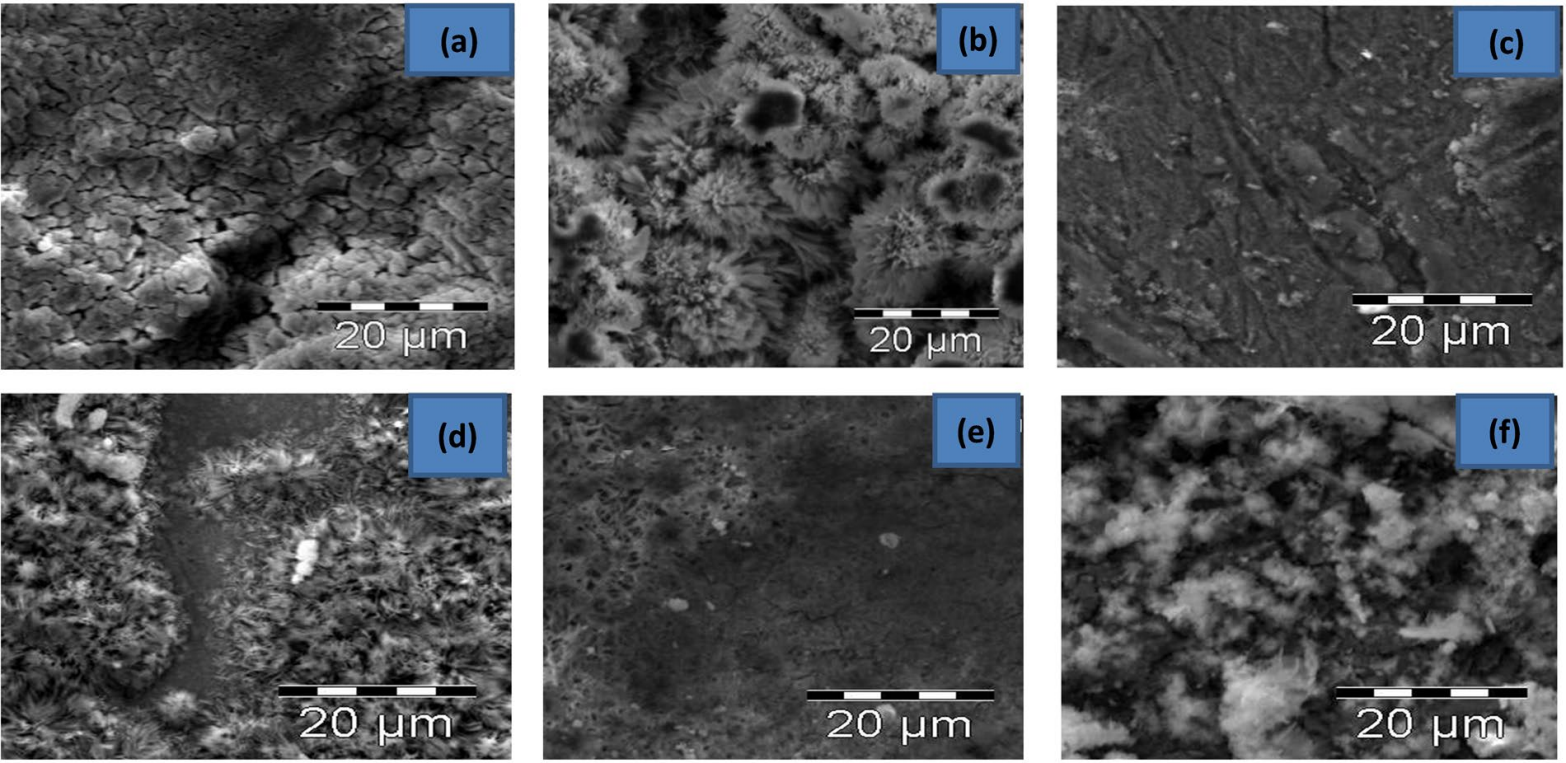
SEM images of mild steel surfaces immersed in 1 M HCl without and with 100 ppm of the studied inhibitors (**a**) blank (**b**) CZ (**c**) THCZ (**d**) HCZ (**e**) EHCZDCA (**f**) DBCZ.

**Figure 8:**
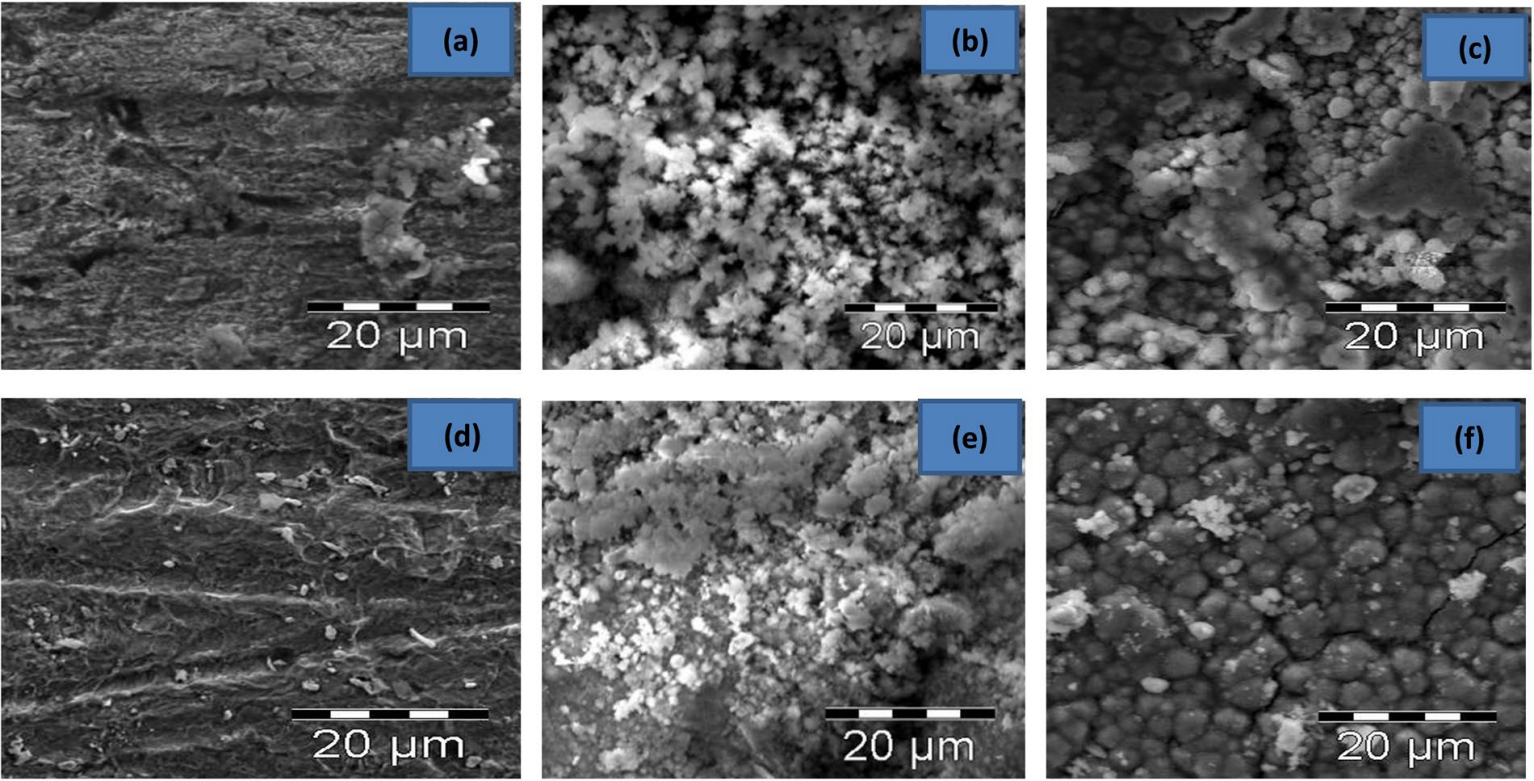
SEM images of mild steel surfaces immersed in SRB culture without and with 100 ppm of the studied inhibitors (**a**) blank (**b**) CZ (**c**) THCZ (**d**) HCZ (**e**) EHCZDCA (**f**) DBCZ.

Figures 7 and 8 respectively show the SEM images of mild steel coupons exposed to the uninhibited and inhibited acid media (for 3 h) and uninhibited and inhibited *D*. *vulgaris* inoculated media (for 9 days). The coupon surfaces was analysed without removing the biological and/or chemical species formed on the coupon surfaces. All the mild steel coupons analysed showed distinctive surfaces after exposure to the respective media.

Figure 7a reveals the SEM micrograph of mild steel surface in 1 M HCl in the absence of the inhibitors, which clearly shows a hugely corroded and damaged surface. In the presence of 100 ppm of the studied CZs, the surface morphologies (Fig. 7b–f) are characterised by strongly localised protective film, which suggests that the studied CZs adsorbed on mild steel surface and prevent it from direct contact with the corrosive acidic ions.

Figure 8a shows the presence of passive film as well as conditioning layers on the mild steel coupons retrieved from the SRB medium (without the inhibitors). In the presence of the studied CZ molecules, the coupon surfaces become more homogeneous than that of SRB medium (Fig. 8b–f). A comparison of the steel surfaces revealed that the mild steel coupons retrieved from the *D*. *vulgaris* inoculated media that contain the inhibitor molecules are covered with protective film of inhibitor molecules, while the one retrieved from the blank *D*. *vulgaris* inoculated medium does not have such film. Yuan *et al*.^26^ reported that the absence of protective surface film tends to facilitate localised attack by sulphide ions, which most SRBs are known to produce. This consequently initiates pitting or crevice corrosion, which is more pronounced for the steel surface immersed in the uninhibited SRB medium as revealed from the large pit depth in Fig. 8a ^50^.

Furthermore, the surfaces of MS coupons exposed to SRB cultures after 9 days incubation with or without 100 ppm CZs are characterized by two distinctive layers (with the exception of Fig. 8a and d) that display both morphological and chemical characteristics of the biofilm (formed) on the MS surface. The dark layer composed of minimal amounts of sulphides and high amounts of carbon-based materials and iron oxides. The light layer on the other hand consists of large amounts of sulphides, carbon-based materials and iron oxides. These findings corroborate with data obtained in the EDX elemental analysis.

#### Energy dispersive X-ray (EDX) study

Elemental analysis using EDX technique was carried out at different points on the corrosion crust for all the mild steel coupons and the results of the analyses are listed in Table 7. It was found that the crystalline particles obtained from coupons immersed in 1 M HCl with or without 100 ppm of CZs consist of iron, chlorine, carbon, oxygen and trace of sulphur.

**Table 7:** EDX elemental constituents (in weight%) of mild steel surface retrieved from the acidic and SRB corrosive media without and with 100 ppm of the inhibitors.

Inhibitors	Fe	Mn	Ca	Cl	S	P	Al	Na	O	C
Acidic media										
1 M HCl Blank	34.55	—	—	34.6	—	—	—	—	23.99	6.86
CZ	55.98	—	—	5.49	—	—	—	—	34.63	3.91
DBCZ	55.32	0.48	—	2.05	0.24	0.07	0.40	0.27	36.24	4.92
HCZ	55.21	—	—	0.16	—	—	—	—	41.28	3.35
THCZ	50.06	0.16	—	0.35	—	1.68	0.09	0.07	22.95	24.64
EHCZDCA	48.89	0.18	—	3.15	1.08	—	0.07	—	39.91	6.71
SRB Culture media										
SRB Blank	76.57	0.15	0.47	—	0.21	0.77	0.10	—	8.90	12.83
CZ	49.92	—	—	—	0.08	—	0.07	—	39.57	10.37
DBCZ	61.15	—	—	0.11	0.07	0.08	0.07	—	24.27	14.23
HCZ	54.52	—	—	—	0.14	0.32	0.07	—	35.54	9.42
THCZ	51.66	—	—	—	0.14	0.42	0.06	–	38.70	9.03
EHCZDCA	50.88	—	—	—	0.19	2.77	0.14	0.38	36.50	9.13

MS surfaces in SRB media were characterized by iron, sulphur, phosphorous, aluminium, oxygen and carbon. The detection of sulphur in all the samples further confirms the presence of SRB. The low level of oxygen on the steel surface retrieved from SRB blank (medium) compared to the presence of the tested CZ molecules may be due to the reducing activities of SRB. SRBs are known for their ability to reduce sulphate and other oxidized (oxygen containing) species in the environment. High oxygen contents observed for the steel in the presence of the inhibitors (in SRB media) might be attributed to the biocidal actions of the CZs on the bacteria, which undermine the reducing activity of SRB.

#### Quantum chemical calculations

In a bid to gain further insights into the donor-acceptor interactions between the studied CZs and mild steel, and to relate the inhibition potentials of the CZs to molecular structures and different substituents on the carbazole moiety, quantum chemical calculations^40^, were performed on the CZs. According to the Fukui’s frontier orbital approximation, donor-acceptor interactions do occur between frontier molecular orbitals (HOMO and LUMO) of interacting/reacting species^32, 51^. A metal has a high tendency of accepting electrons from an electron donor (e.g. an inhibitor molecule) into its lowest unoccupied orbital^51^. Conversely, a metal can donate its HOMO electron into appropriate vacant (LUMO) orbital of the inhibitor molecule^6, 7, 51–54^ for back-bonding. This donor-acceptor relationship between inhibitor molecules and metallic orbitals has been the major molecular and electronic explanation behind the adsorption of inhibitor molecules on metallic surface.

**Figure 9:**
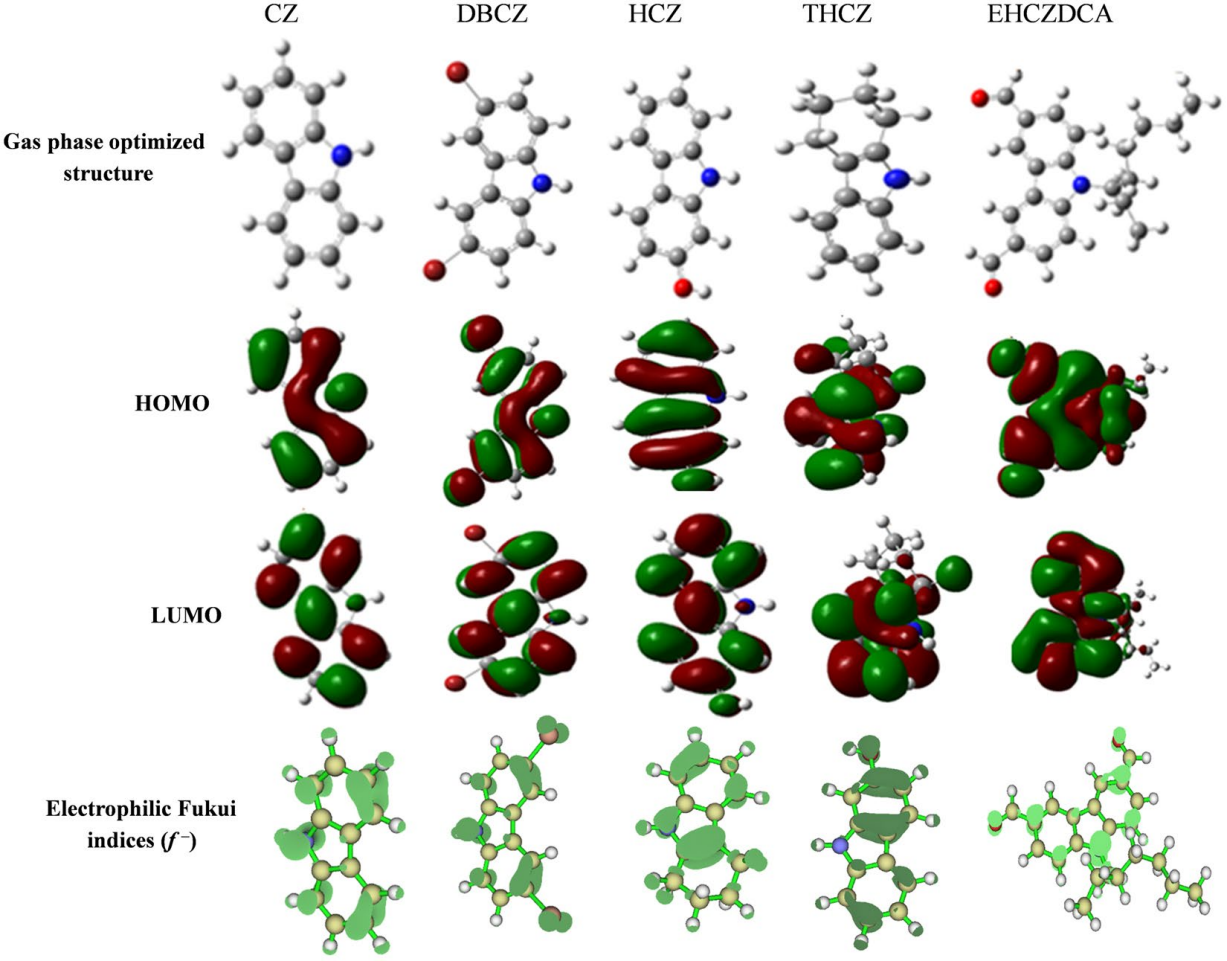
Optimized structure, HOMO, LUMO and the electrophilic Fukui function (*f* ^*−*^, isosurface value = 0.003) for neutral molecules of the studied compounds in the gas phase.

The optimized molecular structures, HOMO and LUMO electronic density distributions and electron density distributions for the electrophilic Fukui function (*f* ^−^) are shown in the graphical images in Fig. 9. The HOMOs of all the molecules are mainly dominated by *π* bonding orbitals and delocalized over the entire aromatic carbazole rings in each molecule. The Br substituents (Br7 and Br15) in DBCZ are also involved in the HOMO.

The HOMO of EHCZDCA is also extended to the ethylhexyl alkyl chain attached to the N1 atom of carbazole ring. The widely distributed HOMO electron density of the studied CZs is an indication of favourable interactions of the molecules with electron deficient metallic orbitals. The LUMO electron densities of all the molecules are distributed over the carbazole rings. The HOMO and LUMO electronic surfaces revealed that the carbazole ring has potential to donate and accept electrons under favourable conditions. This characteristic is favourable to donor-acceptor interactions and might be responsible for the adsorption of the molecules on mild steel surface.

Fukui functions *f* ^+^ and *f* ^−^ are local reactivity indices that are usually employed to analyse the relative susceptibility of the active atomic sites of an inhibitor molecule to electrophilic and nucleophilic attacks, respectively^4^. The preferred site for electrophilic attack is the region/atom in the molecule where the value of *f* ^−^ is the highest^21^. The isosurface density of the Fukui function for electrophilic attack (*f* ^−^) is also shown in Fig. 9. The electron density distributions of the *f* ^−^ revealed that the N-atom of the carbazole ring and the C=C π-electrons centres of the fused aromatic rings are the most susceptible sites for electrophilic attacks in the molecules. The substituent Br atoms in DBCZ, the O-atom of the OH group in HCZ and the C=O functional groups in EHCZDCA respectively are also susceptible to electrophilic attacks.

Some quantum chemical parameters of the studied compounds both in the gas and aqueous phases are listed in Table 8. The energy of the HOMO orbital (E_HOMO_) is a measure of relative tendency of a molecule to donate electrons to an electron accepting specie^6, 7, 51–54^. The results in Table 8 show that THCZ has the highest value of HOMO energy (E_HOMO_) in the gas phase, which implies highest tendency to donate electrons to an electron deficient site. This suggests that THCZ has the highest tendency to adsorb onto mild steel surface and the observation is in agreement with its highest inhibition efficiency observed from the experiments. The LUMO energy (E_LUMO_) is a measure of the tendency of a molecule to accept electron(s) from electron rich specie.

**Table 8:** Selected quantum chemical parameters for the studied carbazoles.

Inhibitor	E_HOMO_ (eV)	E_LUMO_ (eV)	ΔE (eV)	Dipole moment	η	σ	µ	ω	MP	MV (Å^3^)
Gas phase										
CZ	−5.76	−1.09	4.67	1.66	2.33	0.43	−3.43	2.52	55.17	184
DBCZ	−6.02	−1.62	4.40	4.40	2.20	0.46	−3.83	3.33	58.19	220
HCZ	−5.67	−0.97	4.70	2.89	2.35	0.43	−3.32	2.34	55.74	191
THCZ	−5.38	−0.38	4.00	2.54	2.50	0.40	−2.89	1.66	55.81	193
EHCZDCA	−6.35	−2.10	4.25	7.90	2.12	0.47	−4.23	4.21	70.66	373.51
Aqueous phase										
CZ	−5.84	−1.21	4.63	2.32	2.32	0.43	−3.53	2.69	—	—
DBCZ	−5.96	−1.56	4.41	2.20	2.20	0.45	−3.76	3.21	—	—
HCZ	−5.80	−1.08	4.72	2.36	2.36	0.42	−3.44	2.51	—	—
THCZ	−6.26	−2.22	4.04	2.02	2.02	0.50	−4.24	4.45	—	—
EHCZDCA	−5.51	−0.56	3.85	2.48	2.48	0.40	−3.04	1.86	—	—

Molecules with low *E*_LUMO_ values tend to accept electrons easily^6, 7, 51–54^. Though, EHCZDCA has a relatively low E_HOMO_, its very low E_LUMO_ offers it a better chance of accepting charges in a favourable retro-donation/back-bonding formation during donor-acceptor interactions with the metal. The very low value of E_LUMO_ of EHCZDCA might be responsible for its relatively higher inhibition efficiency compared to CZ, DBCZ and HCZ.

The frontier molecular orbitals energy gap, Δ*E* (Δ*E* = *E*_*LUMO*_ = *E*_*HOMO*_) is often used to characterize the chemical reactivity and kinetic stability of molecules^6, 55^. Murulana *et al*.^6^ posited that molecules with large value of Δ*E* are highly stable (i.e., they have low reactivity to chemical species) whilst molecules with small value of Δ*E* have a high reactivity. The results reported in Table 8 show EHCZDCA has the smallest Δ*E* value and therefore corresponds to the most reactive compound. This is supportive of the high inhibition efficiency of EHCZDCA especially at 500 ppm compared to CZ, DBCZ and THCZ.

Dipole moment is a descriptor of the polarity of molecules^6, 32^. Abdallah *et al*.^32^ alluded that dipole moment is an indicator of the electronic distribution in a molecule and is one of the properties used to rationalize molecular structure.

There is however, lack of general consensus on the correlation between the dipole moment and corrosion inhibition efficiency^56, 57^. There is an opinion that high dipole moment favours high inhibition efficiency^58^, while a dissenting opinion infers conversely^59^. There is no general trend of relationship between the dipole moments of the studied CZs and experimental inhibition efficiencies. Although, CZ with the least dipole moment has the least inhibition efficiency, while EHCZDCA with considerably high inhibition efficiency was found to have very high dipole moment. This observation is somewhat in agreement with the assumption that high moment enhances dipole-dipole interactions between the inhibitor molecules and charged metal surface and hence favours inhibition efficiency^58, 59^.

Molecular volume (MV) is a measure of the contact surface between the corrosion inhibitor molecule and metal surface^6^. The corrosion inhibition efficiency is usually proportional to the fraction of the surface covered by the adsorbed inhibitor molecule. Murulana *et al*.^6^, however, suggested that this is not always the case, considering the fact that corrosion inhibition is often influenced by multiple factors that are interdependent. The highest value of MV for EHCZDCA in the present study supports its high inhibition efficiency compared to DBCZ, HCZ and CZ.

Global (chemical) hardness (*η*) and softness (*σ*) have all been used as molecular descriptors of reactivity and selectivity^6, 60, 61^. The relationship between these quantum chemical descriptors and corrosion inhibition is often interpreted based on the Lewis theory of acid and bases and Pearson’s concept of hard and soft acids and bases^6, 32^. In this concept, a hard molecule is said to have a large Δ*E* value, while a soft molecule has a small Δ*E* value. Adsorption usually occurs at the region of the molecule where *σ* has the highest value^6^. The order across structures in the *σ* values as reported in Table 8, is such that EHCZDCA > DBCZ > [CZ] ≈ HCZ > THCZ, which also suggests that EHCZDCA is the most reactive compound.

Global electronic chemical potential (*µ*) is often reported as electronegativity (χ) in most previous studies^62, 63^. According to Wang *et al*.^62^ and Udhayakala *et al*.^63^, electronegativity is often described as the negative of global electronic chemical potential. The values of *µ* reported in Table 8 show that the order of the *µ* values is THCZ > HCZ > CZ > DBCZ > EHCZDCA.

Electrophilicity index (*ω*) is another global reactivity index that is often used for the prediction of the direction of a corrosion inhibition process^60, 61^. A high electrophilicity value describes a good electrophile while a small electrophilicity value describes a good nucleophile. The order across structures in the *ω* values as reported in Table 8, is such that EHCZDCA > DBCZ > CZ > HCZ > THCZ, which is in partial agreement with the experimental inhibition efficiencies.

### Molecular dynamics simulation

**Figure 10:**
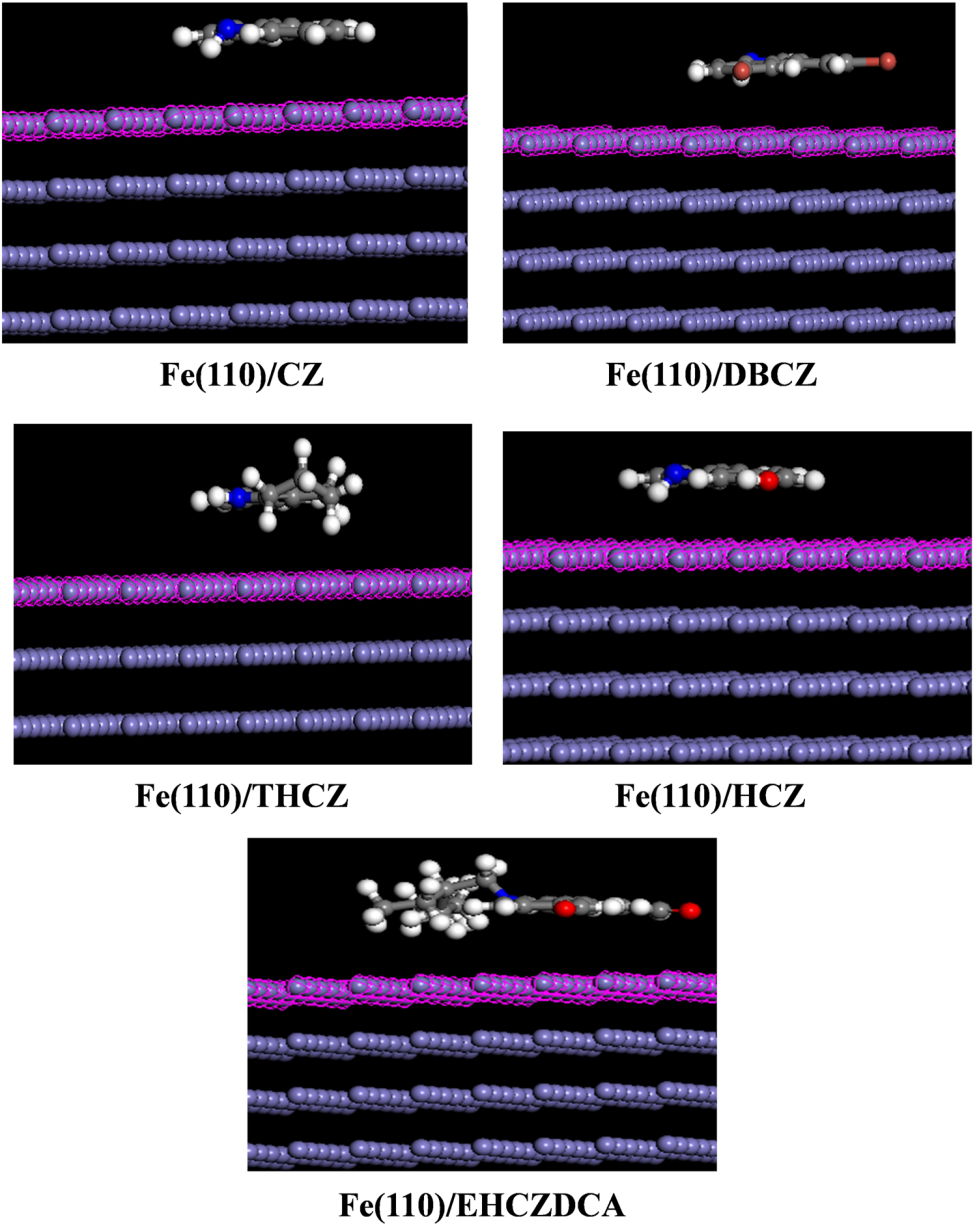
Equilibrium configurations of Fe(110)/inhibitor systems for the studied molecules.

The adsorption of the studied inhibitor molecules on mild steel surface was simulated by modelling the interactions between the inhibitor molecules and Fe(110) crystal surface. The equilibrium configurations of the simulated systems are shown in Fig. 10, while the relevant energy parameters of the systems are listed in Table 9.

**Table 9:** Energy parameters (kcal/mol) associated with the adsorption of the inhibitor molecules on Fe(110).

System	Adsorption energy	Rigid adsorption energy	Deformation energy
Fe(110)/CZ	−91.25	−91.65	0.40
Fe(110)/DBCZ	−109.89	−110.42	0.53
Fe(110)/THCZ	−93.86	−94.64	0.79
Fe(110)/HCZ	−95.43	−95.79	0.36
Fe(110)/EHCZDCA	−178.18	−185.07	6.89

The results (Fig. 10) showed that the moieties containing the aromatic rings and heteroatoms in the inhibitor molecules adsorbed on Fe(110) surface in a near flat orientation, which ensure optimum interactions of the inhibitor molecules with the metallic surface. The highest magnitudes of adsorption and deformation energies were obtained for the Fe(110)/EHCZDCA (Table 9), which suggests that EHCZDCA has the strongest interaction with Fe(110) surface.

## Conclusions

The inhibitive effects of five carbazole derivatives (CZs) on mild steel corrosion and bio-corrosion in 1 M HCl and microbial environments were investigated using electrochemical techniques, weight loss measurements, scanning electron microscopy (SEM) and energy dispersive x-ray (EDX) techniques. Theoretical quantum chemical calculations and molecular dynamic simulation studies were used to corroborate experimental findings. The following conclusions can be drawn from the results:

1. Potentiodynamic polarization measurements revealed that the studied CZs are mixed-type corrosion inhibitors with predominantly cathodic inhibitive effects.
2. Both electrochemical and weight loss results showed that all the compounds inhibit both the acid (1 M HCl) induced corrosion and microbial corrosion and their inhibition efficiencies increase with increase in concentration.
3. The highest inhibition efficiencies were recorded for THCZ and EHCZDCZ in 1 M HCl, while CZ showed the highest protection efficiency against MIC.
4. The studied inhibitor molecules inhibit mild steel corrosion in 1 M HCl by adsorbing on the steel surface and form protective film. Their adsorption obeys Langmuir isotherm and occur mainly via physisorption.
5. SEM images and EDX analyses also revealed that the studied compounds adsorbed on mild steel surface and form protective film that shield the surface from direct effect of corrosion in acidic and SRB media.
6. Molecular quantum chemical calculations showed that the reactive sites in the inhibitor molecules are mainly the N-atom of the carbazole ring, the pi-electron centres, and the other substituent heteroatoms (Br in DBCZ, O-H in HCZ, and C = O in EHCZDCA).
7. Molecular dynamic simulations revealed that the studied molecules have strong interactions with Fe surface.
